# Papain-like cysteine proteases are required for the regulation of photosynthetic gene expression and acclimation to high light stress

**DOI:** 10.1093/jxb/erab101

**Published:** 2021-03-04

**Authors:** Sarah Alomrani, Karl J Kunert, Christine H Foyer

**Affiliations:** 1 School of Biosciences, University of Birmingham, Edgbaston, Birmingham, UK; 2 Centre for Plant Sciences, School of Biology, Faculty of Biological Sciences, University of Leeds, Leeds, UK; 3 Department of Plant and Soil Sciences, Forestry and Agricultural Biotechnology Institute, University of Pretoria, Pretoria, South Africa; 4 Western Sydney University, Australia

**Keywords:** Acclimation, carbon assimilation, chloroplast to nucleus signalling, D1, GUN pathway, high light, photosynthetic gene expression, protein turnover, Rubisco

## Abstract

Chloroplasts are considered to be devoid of cysteine proteases. Using transgenic Arabidopsis lines expressing the rice cystatin, oryzacystatin I (OC-I), in the chloroplasts (PC lines) or cytosol (CYS lines), we explored the hypothesis that cysteine proteases regulate photosynthesis. The CYS and PC lines flowered later than the wild type (WT) and accumulated more biomass after flowering. In contrast to the PC rosettes, which accumulated more leaf chlorophyll and carotenoid pigments than the WT, the CYS lines had lower amounts of leaf pigments. High-light-dependent decreases in photosynthetic carbon assimilation and the abundance of the Rubisco large subunit protein, the D1 protein, and the phosphorylated form of D1 proteins were attenuated in the CYS lines and reversed in the PC lines relative to the WT. However, the transgenic lines had higher amounts of *LHC*, *rbcs*, *pasbA*, and *pasbD* transcripts than the WT, and also showed modified chloroplast to nucleus signalling. We conclude that cysteine proteases accelerate the reconfiguration of the chloroplast proteome after flowering and in response to high-light stress. Inhibition of cysteine proteases, such as AtCEP1, slows chloroplast protein degradation and stimulates photosynthetic gene expression and chloroplast to nucleus signalling, enhancing stress tolerance traits.

## Teaser

Chloroplasts nominally lack cysteine proteases but chloroplast protein turnover and gene expression were modified in Arabidopsis plants expressing a cysteine protease inhibitor in the chloroplasts or cytosol.

## Introduction

While plant cysteine proteases fulfil many important functions in plants, they have never been shown to localize to chloroplasts, except in barley (Frank *et al.*, 2019). The major chloroplast protease families (Clp, FtsH, and DegP) are constitutively expressed and fulfil roles in protein quality control and the maintenance of photosynthesis rather than large-scale remodelling of the chloroplast proteome. Plastid functions are regulated by the ubiquitin–proteasome system (UPS) through the action of the SP1 ubiquitin E3 ligase and the SP2 channel protein that are localized on the outer chloroplast envelope membrane (Ling *et al.*, 2012, 2019). SP1 and SP2 target the plastid protein import machinery [TOC (translocon of the outer membrane) proteins] for degradation in a novel proteolytic pathway, which is called chloroplast-associated protein degradation (CHLORAD). This pathway regulates the TOC machinery to facilitate reconfiguration of the plastid proteome, which is essential during stages of plant development that involve the interconversion of different plastid types, for example reconfiguration of plastid proteins when chloroplasts are converted into gerontoplasts during leaf senescence (Ling *et al.*, 2012, 2019). Moreover, CHLORAD has an additional role in abiotic stress responses (Ling *et al.*, 2019).

Cysteine proteases can be divided into 14 superfamilies, each with a catalytic triad or dyad in a different structural fold (Rawlings *et al.*, 2018). Small ubiquitin-like modifier (SUMO) cysteine proteases regulate the SUMO cycle by cleaving SUMO from SUMOylated proteins, providing specificity to which proteins become SUMOylated and generating free SUMO (Botha *et al.*, 2017; Morrell and Sadanandom, 2019). The papain-like cysteine proteases (PLCPs) are classified into clan CA because of the structural similarity of their conserved catalytic residues to papain (Rawlings *et al.*, 2018). Thirty-one genes encoding PLCPs have been identified in the Arabidopsis genome. These enzymes play a key role in the regulation of protein turnover, particularly in leaf senescence and the recycling of nutrients from the senescing leaves to growing leaves and reproductive organs (Díaz-Mendoza *et al.*, 2014; Liu *et al.*, 2018). The activities of PLCPs are tightly controlled via autocatalytic post-translational modifications, as well as by cystatins and serpins (van der Linde *et al.*, 2012; Lampl *et al.*, 2013). PLCPs contain the conserved catalytic triad (Cys, His, Asn). They perform a nucleophilic attack of the thiol group at the substrate C-terminus where His acts as a proton acceptor (base) for the catalytic Cys, and Asn plays an important role for the orientation of His (Rawlings *et al.*, 2018).

Cystatins are proteins that contain a Gln–Xaa–Val–Xaa–Gly motif in the centre of the polypeptide chain (where Xaa is any amino acid), a Pro–Trp (or Leu–Trp) dipeptide motif in the C-terminal region, and a conserved Gly residue in the N-terminus (Benchabane *et al.*, 2010). Cystatins bind to the active site of their target cysteine proteases and inhibit enzyme activity in an irreversible manner. Studies of *in vivo* interactions between a cystatin and cathepsin L-like cysteine protease showed co-localization to the endoplasmic reticulum and the Golgi complex (Martinez *et al.*, 2009). The functions of PLCPs and phytosystatins have been intensively studied, particularly in relation to plant development, stress responses, and pest control (Van der Hoorn, 2008; Kunert *et al.*, 2015). In particular, oryzacystatin I (OC-I), which is a small protein of 120 amino acids that is a competitive inhibitor of PLCPs (Benchabane *et al.*, 2010), has been extensively studied in a range of transgenic plants including tobacco (Van der Vyver *et al.*, 2003; Prins *et al.*, 2008), soybean, and Arabidopsis (Quain *et al.*, 2014). These studies showed that cysteine proteases play a role in chloroplast protein turnover (Prins *et al.*, 2008).

Despite intensive efforts, molecular–genetic and biochemical studies have failed to identify chloroplast-localized cysteine proteases in Arabidopsis and plant species. However, a barley C1A family cysteine protease, called HvPAP14, was recently found in the thylakoid lumen of the chloroplasts, as well as the endoplasmic reticulum and vesicular bodies (Frank *et al.*, 2019). HvPAP14 is involved in the degradation of the light-harvesting Chl *a*/*b*-binding proteins and the large subunit of Rubisco (Frank *et al.*, 2019). There is only one other report which has provided evidence of cysteine protease activity in the thylakoid lumen (Sokolenko *et al.*, 1997).

While a small number of proteins are encoded by chloroplast genes, most of the photosynthetic proteins are encoded by the nuclear genome. This requires the coordinated regulation of the transcriptional activity of both genomes, a process that involves bi-directional communication from the nucleus to the chloroplasts (anterograde signalling), and from chloroplasts to the nucleus (retrograde signalling). Retrograde signals provide information concerning the metabolic and energy state of the chloroplasts that facilitates appropriate responses to environmental cues (Chan *et al.*, 2016). Mature chloroplasts produce a wide range of signalling molecules that act as retrograde signals, such as tetrapyrroles, that affect the expression of plastid redox-associated nuclear genes (PHANGs) in the nucleus in response to environmental cues (Chan *et al.*, 2016). Retrograde signalling between the chloroplasts and nuclei has been intensively studied using the carotenoid synthesis inhibitor norflurazon (NF) and/or the plastid translation inhibitor, lincomycin (LINCO). These inhibitors cause oxidation of the cytosol and nuclei, leading to the repression of nuclear genes encoding components of the photosynthetic electron transfer chain such as the light-harvesting Chl *a*/*b*-binding complex proteins and plastocyanin (Grubler *et al.*, 2017; Karpinska *et al.*, 2017). Screens using these inhibitors have revealed the plastid-localized genomes uncoupled (GUN) retrograde signalling pathway (Shimizu *et al.*, 2019).

This study was conducted to test the hypothesis that cysteine proteases play a role in reconfiguration of the chloroplast proteome and photosynthesis in response to changes in environmental stimuli such as light intensity. In order to test whether cysteine protease activities localized within chloroplasts play a role in this regulation, we prepared transgenic Arabidopsis lines expressing the rice cystatin OC-I targeted to the chloroplast stroma. We compared shoot traits and photosynthetic properties in transgenic lines with OC-I-dependent inhibition of cysteine proteases targeted to the chloroplasts with those where OC-I was expressed with no intracellular targeting and hence the product of the transgene is presumed to be localized is the cytosol. We show that the growth and shoot phenotypes of transgenic lines expressing OC-I in the chloroplasts (PC lines) or in the cytosol (CYS lines) were modified in the OC-I-expressing lines compared with wild-type (WT) plants. In addition, evidence is presented showing that exposure to high light (HL), which is widely accepted as a stress that triggers local and systemic signalling to regulate stomatal closure and activate the expression of defence-related genes (Karpinski *et al.*, 1999; Devireddy *et al.*, 2018), leads to a substantial increase in leaf protease activities, particularly cysteine protease activities in the WT but not in the PC and CYS lines. The absence of HL-induced increases in leaf cysteine protease activities protects photosynthesis against HL-induced inhibition by limiting the decreases in the abundance of the Rubisco large subunit protein, the D1 protein, and the phosphorylated form of D1 that were observed in the WT plants. Moreover, photosynthetic gene expression was altered in the PC and CYS lines, in a manner that suggests that cysteine proteases are involved in chloroplast to nucleus signalling.

## Materials and methods

### Production of transgenic Arabidopsis lines expressing OC-I targeted to the chloroplasts

The OC-I gene was cloned as a *Sac*I–*Xba*I fragment into the plasmid pLBR19 to create plasmid pLBRCys-1. The pLBRPRKCys-1 plasmid contained the OC-I gene with expression under the control of the double 35S promoter of the *Cauliflower mosaic virus* (CaMV), an ampicillin resistance gene, and a CaMV terminator. The pLBRPRKCys-1 plasmid also contained the sequence encoding the signal peptide of the stomal enzyme phosphoribulokinase (PRK), leading to targeting of the OC-I protein in the chloroplast stroma. Transgenic plants expressing the plasmid were prepared by the floral dip method from the *Agrobacterium tumefaciens* strain GV3101. Successive generations were screened for herbicide resistance and expression of the transgene. T_4_ generation plants that were homozygous for the transgene were used in the following experiments.

### Plant material and growth conditions

Seeds of WT *Arabidopsis thaliana* (L.) and six independent transgenic lines overexpressing OC-I either with the chloroplast PRK targeting sequence (PC2, PC7, and PC9) or without a targeting sequence for localization in the cytosol (CYS1, CYS3, and CYS4; Makgopa, 2014; Quain *et al.*, 2014) were used in the following experiments. Unless otherwise stated below or in the figure legends, each experiment involved six T_4_ generation plants from each line, together with six WT plants. Plants were grown in 10 cm pots containing potting compost for up to 12 weeks in controlled-environment chambers with a 16 h/8 h day/night photoperiod (light intensity of 250 μmol m^–2^ s^–1^) at 20 °C, 60% humidity. All genotypes were grown at the same time in the same controlled-environment chambers.

### High-light treatments

The transgenic and WT Arabidopsis plants were grown in compost in controlled-environment chambers under a moderate light (LL) intensity (250 μmol m^–2^ s^–1^) for 5 weeks. Thereafter, before the start of the next photoperiod, half of the plants were transferred to HL (800 μmol m^–2^ s^–1^) supplied by light-emitting diodes (LEDs) in the controlled-environment chambers and subjected to HL for the whole 8 h photoperiod, while the remaining half were maintained for 8 h at 250 μmol m^–2^ s^–1^. Leaf samples were collected between 7 h and 8 h into the 8 h photoperiod. The metabolism of harvested material was arrested immediately by immersion in liquid nitrogen. Thereafter, frozen samples were stored at –80 °C for further analysis. Six plants per line were used in each experiment. All lines were grown together in each experiment and subjected to the different light treatments simultaneously. Each experiment was repeated at least three times.

### Shoot growth analysis

At the time points indicated in the figure legends, the WT, PC, and CYS lines were harvested and separated into shoots and roots. The shoots were immediately weighed and then placed in an oven at 80 °C for 2 d to determine shoot dry biomass.

The time of flowering—the point at which the plants produce flowering stems—was also recorded.

### Leaf pigment analysis

Leaf chlorophyll and protein contents were determined in leaf samples that had been ground in liquid nitrogen. Pigments extracted in 96% (v/v) ethanol were determined according to Lichtenthaler and Wellburn (1983). The soluble protein content was determined according to the method of Bradford (1976).

Photosynthetic pigments were extracted from frozen leaf samples that had been ground in liquid nitrogen by the addition of 80% (v/v) acetone and were measured as described by Lichtenthaler (1987).

### Leaf protein analysis

Leaf proteins were extracted using protein extraction buffer (AS08 300, Agrisera). Protein concentrations (mg g^–1^) were determined using the Pierce Microplate BCA Protein Assay Kit (Thermo Scientific) employing a Fluostar Omega plate reader (BMG Labtech GmbH).

### Western blot analysis

Protein samples (10 µg) were separated on 4–20% Mini-PROTEAN^®^ TGX™ pre-cast gels (Bio-Rad). A typical example of one Coomassie Brilliant Blue-stained gel is shown in Supplementary Fig. S1. After electrophoresis, proteins were transferred to (0.45 µm) nitrocellulose membranes (Amersham 10600003). Individual membranes were then incubated with the following primary antibodies at a 1:10 000 dilution: Rubisco large subunit (RbcL) form I and form II (AS03 037, Agrisera, Sweden), the PSII D1 protein (AS05 084, Agrisera, Sweden), and PsbA (D1) and the phosphorylated form of D1 (AS13 2669, Agrisera, Sweden). A typical example of an individual Western blot is shown in Supplementary Fig. S1, together with a typical loading control gel.

### Total protease activity and cysteine protease activity

Protease activities were determined using the Abcam Assay Kit (ab111750), which incorporates fluorescein isothiocyanate (FITC)-labelled casein as a general protease substrate. The FITC–casein substrate is broken down into small peptides by the leaf proteases, resulting in a decrease in fluorescence quenching. The fluorescence of peptide fragments was estimated at an excitation/emission (Ex/Em) wavelength of 485/530 nm. Cysteine protease activities were determined by pre-incubation of the samples with the specific cysteine protease inhibitor E-64 (5 mM; Sigma).

### Photosynthetic gas exchange measurements

All genotypes were grown in pots together in the same controlled-environment chambers under LL (250 μmol m^–2^ s^–1^) for 5 weeks. Thereafter, half of the plants were transferred to HL (800 μmol m^–2^ s^–1^) for 8 h, while the remaining half were maintained for 8 h at 250 μmol m^–2^ s^–1^. Photosynthetic gas exchange measurements were performed on plants in the controlled-environment chambers between 7 h and 8 h into the photoperiod under each irradiance.

Measurements were performed on fully expanded leaves essentially as described by Soares *et al.* (2008) using a LICOR portable photosynthetic gas exchange system (Model LI-6400XT).

Photosynthetic CO_2_ assimilation rates were measured on whole leaves at 420 ppm CO_2_, 20 °C, at an irradiance of 400 µmol m^–2^ s^–1^. Leaves were allowed to reach steady-state gas exchange conditions by maintaining the leaves for 10 min in the light and CO_2_ conditions of the chamber prior to measurements. All experiments were conducted at 60% relative humidity. Vapour water deficits were kept constant throughout the assays.

### Chl *a* fluorescence quenching measurements

Chl *a* fluorescence quenching measurement were performed on intact leaves using the LICOR portable photosynthetic gas exchange system (Model LI-6400XT) equipped with the leaf chamber fluorometer between 7 h and 8 h into the photoperiod under each irradiance. Plants were dark adapted for 45 min before each procedure (Plumb *et al.*, 2018). *F*_m_ and *F*_o_, the maximum and minimum yields of fluorescence, respectively, were measured in dark-adapted leaves. Dark-adapted *F*_v_/*F*_m_, Φ _PSII_, and non-photochemical quenching (NPQ) were calculated as *F*_v_/*F*_m_=(*F*_m_−*F*_o_)/*F*_m_, Φ _PSII_=(*F*_m_′−*F*_s_)/*F*_m_′, and NPQ=(*F*_m_−*F*_m_′)/*F*_m_′, respectively.

### Norflurazon and lincomycin treatments

Seeds of the transgenic *A. thaliana* and WT lines were grown in the absence or the presence of inhibitors using a standard lab protocol (Karpinska *et al.*, 2017). CYS, PC, and WT seeds were grown on half-strength Murashige and Skoog medium containing 0.1 g l^−1^ myoinositol, 10 g l^−1^ sucrose, and 0.5 g l^−1^ MES buffer (pH 5.7) supplemented with either NF (5 µM), LINCO (500 µM), or ethanol as a control. Seedlings were grown for 7 d in a controlled-environment chamber with a 16 h/8 h day/night photoperiod and a daytime irradiance of 100 μmol m^–2^ s^–1^. Seedlings were then harvested for RNA extraction and further analysis. Each experiment, which involved 20 seedlings per line, was repeated at least three times.

### Quantitative real-time reverse transcription–PCR (qRT–PCR)

For these analyses, plants were grown on compost in controlled-environment chambers at 250 μmol m^–2^ s^–1^ for 5 weeks. Thereafter, half of the plants were transferred to 800 μmol m^–2^ s^–1^) for 8 h. Six plants per line were used in each experiment, and each experiment was repeated three times. Leaf samples were collected between 6 h and 8 h into the photoperiod. Metabolism was arrested immediately in liquid nitrogen, and samples were stored at –80 °C for analysis. RNAs were extracted from leaves using the Spectrum™ plant total RNA kit protocol. Following synthesis of cDNA using the QuantiTect Reverse Transcription Kit (Qiagen), qRT–PCR was performed using a Quantifast SYBR Green RT-PCR Kit (Qiagen). The expression of the genes of interest was performed using the primer sequences given in Supplementary Table S1 relative to two (actin and SAND) control transcripts.

### Statistical analysis

All datasets were analyzed by a two-ANOVA. If an independent variable (e.g. light) had a significant effect on the response (*P*≤0.05), Tukey’s multiple comparison test was used to compare means between genotypes, while Sidak’s multiple comparison test was applied to compare light effects within each genotype. For datasets which were normally distributed, Tukey’s HSD (honestly significant difference) was used as a post-hoc test at a stringency level of *P* <0.05. Asterisks indicate statistical significance as follows: *P<0.05, **P<0.01, ***P<0.001, and ****P<0.0001. Each measurement in the phenotypic analysis involved 24 plants per line, and three replicates were performed for each experiment. All statistical analysis was performed using SPSS v.13 for windows (Statistical Package for Social Sciences, Chicago, IL, USA).

## Results

### Shoot phenoptyes

**Fig. 1.**
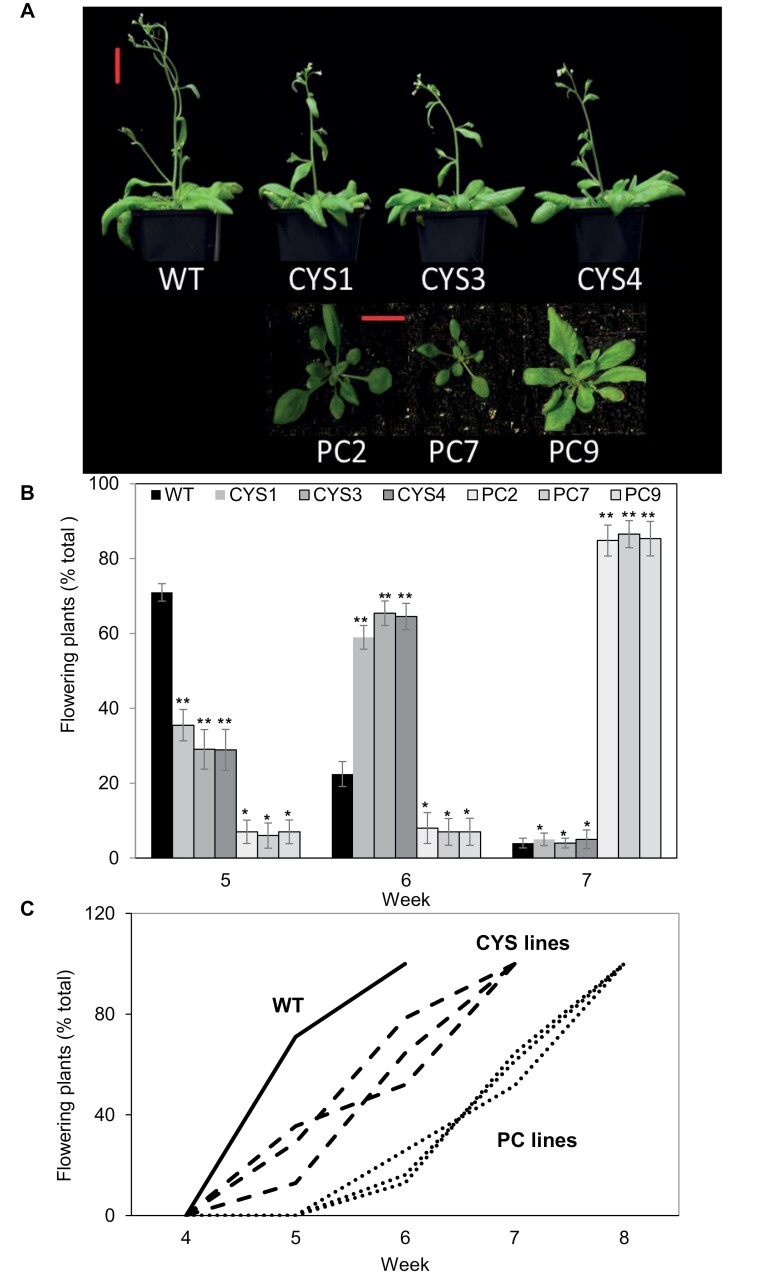
The phenotype of wild-type *A. thaliana* (WT) and six independent transgenic lines expressing OC-I either in the cytosol (CYS1, CYS3, and CYS4) or in the chloroplast (PC2, PC7, and PC9). Representative images of the shoot phenotypes of 6-week-old plants (A), the number of flowering plants per week (B), and flowering time trends for the different lines (C). *n*=24 plants per line. Scale bar=3 cm.

The plants expressing OC-I without a targeting sequence (CYS1, CYS3, and CYS4) had a similar shoot phenotype to the WT at 6 weeks (Fig. 1A). However, the transformed lines expressing OC-I in the chloroplast (PC2, PC7, and PC9) were visibly smaller than the WT at 6 weeks. Moreover, the WT plants had flowered at 6 weeks but the PC lines were without flowers (Fig. 1A). All of the transgenic lines flowered later than the WT (Fig. 1B), the PC lines showing the greatest delay in flowering (Fig. 1C).

**Fig. 2.**
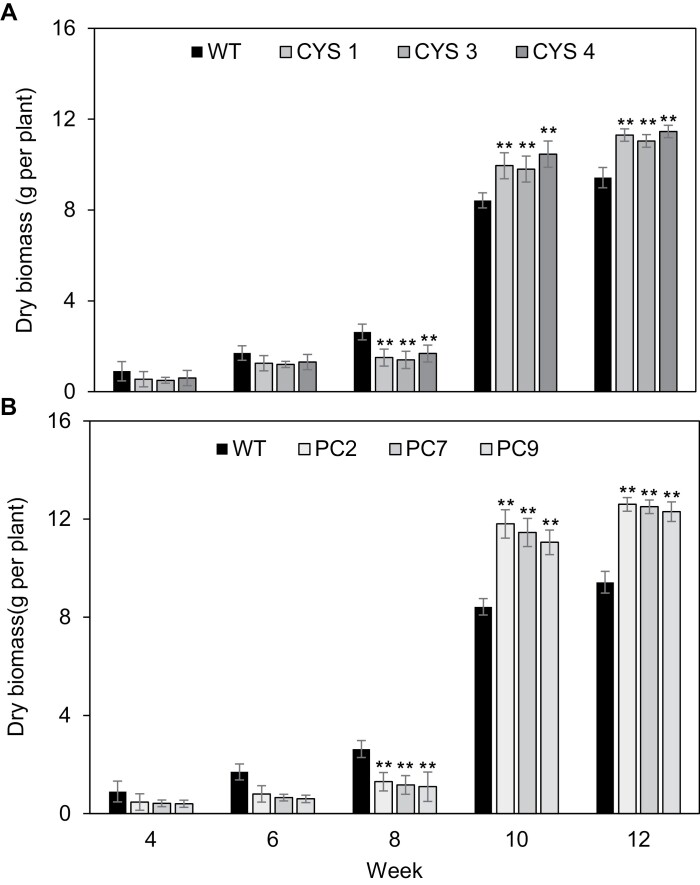
A comparison of shoot biomass (dry mass per plant) in the *A. thaliana* plants expressing OC-I either in the cytosol (A: CYS1, CYS3, and CYS4) or in the chloroplast (B; PC2, PC7, and PC9) compared with the WT plants at 4, 6, 8, 10, and 12 weeks after sowing. Bars show the means ±SD (*n*=24 plants per line). Statistical significance is indicated by asterisks: *P*-value ≤0.05, **P-value ≤0.01.

**Fig. 3.**
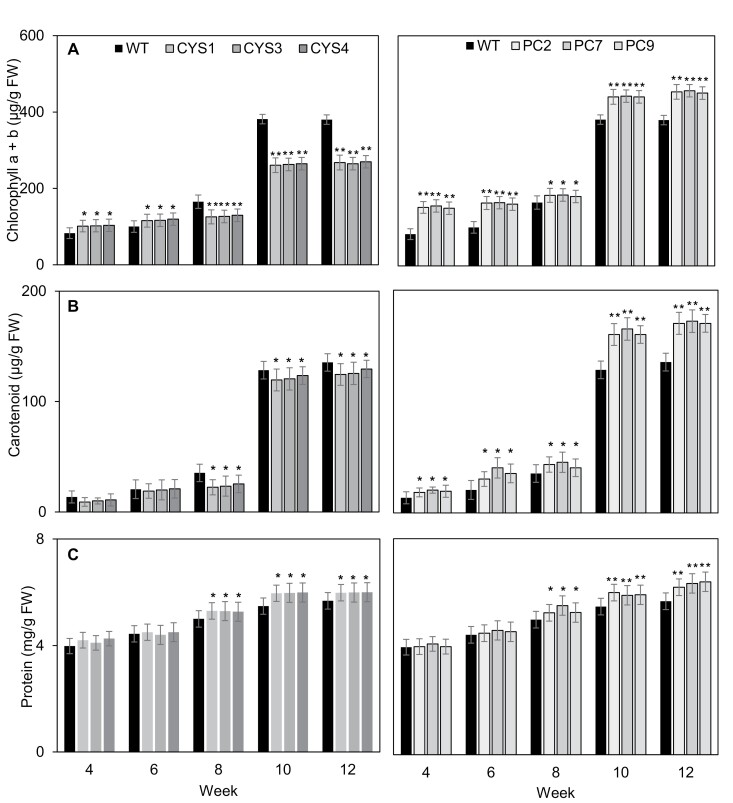
A comparison of the (A) chlorophyll and (B) carotenoid pigment, and (C) soluble protein amounts in the leaves of *A. thaliana* plants expressing OC-I either in the cytosol (CYS1, CYS3, and CYS4) or in the chloroplast (PC2, PC7, and PC9) compared with the wild-type (WT) plants at 4, 6, 8, 10, and 12 weeks after sowing. Bars represent means ±SD (*n*=24 plants per line). Statistical significance as determined by *t*-testing is indicated by asterisks: *P*-value ≤0.05, **P-value ≤0.01.

The CYS (Fig. 2A) and PC (Fig. 2B) lines had a lower shoot biomass than the WT during vegetative development but, after flowering, shoot biomass was greater than that of the WT. The CYS leaves had similar amounts of chlorophyll (Fig. 3A) and carotenoid (Fig. 3B) pigments and protein (Fig. 3C) to the WT during vegetative development. However, after flowering, the CYS leaves had significantly lower amounts of chlorophyll (Fig. 3A) and carotenoid (Fig. 3B) pigments than the WT, but higher amounts of leaf protein (Fig. 3C). In contrast to the CYS lines, the PC lines had higher amounts of chlorophyll (Fig. 3A) and carotenoid (Fig. 3B) pigments than the WT throughout development. After flowering, the PC leaves accumulated greater amounts of leaf protein (Fig. 3C) than the WT.

### Effects of high light on photosynthesis

**Fig. 4.**
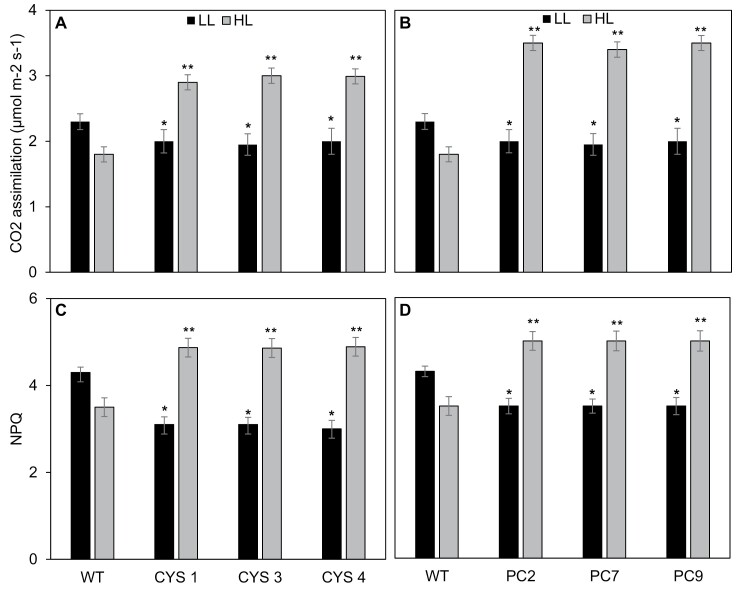
The effects of high light (HL) on photosynthetic CO_2_ assimilation rates and on the non-photochemical quenching of Chl *a* fluorescence (NPQ) in the leaves of CYS (A and C) and PC (B and D) lines compared with the WT. The CYS, PC, and WT plants were grown under moderate light (LL: 250 μmol m^–2^ s^–1^) for 5 weeks. Thereafter, half of the plants were transferred to HL (800 μmol m^–2^ s^–1^) for 8 h, while the remaining half were maintained for 8 h at 250 μmol m^–2^ s^–1^). Photosynthetic CO_2_ assimilation and NPQ were measured at the end of the photoperiod in all lines. Bars represent means ±SD (*n*=24 plants per line). Statistical significance is indicated by asterisks: *P-value ≤0.05.

Photosynthetic CO_2_ assimilation rates were slightly lower in the CYS and PC lines than in the WT under the growth light intensity (250 μmol m^–2^ s^–1^) conditions (Fig. 4A, B). However, after transfer to HL (800 μmol m^–2^ s^–1^), photosynthesis rates were significantly decreased in the WT plants. In contrast, photosynthetic CO_2_ assimilation rates were increased in the CYS (Fig. 4A) and PC lines (Fig. 4B) under HL compared with LL, and were significantly higher in the transgenic lines than in the WT under HL conditions. Photosynthesis was 60% higher in the PC lines than in the WT under HL conditions (Fig. 4B).

The calculated values for the flexible NPQ mechanisms of the photosynthetic electron transport system that allow the dissipation of excess light energy as heat were lower in the CYS and PC lines than in the WT under LL (Fig. 4C, D). While exposure to HL slight decreased NPQ values in the WT, NPQ levels were significantly increased in the CYS and PC lines under HL (Fig. 4C, D).

### Effects of high light on leaf protease activities

**Fig. 5.**
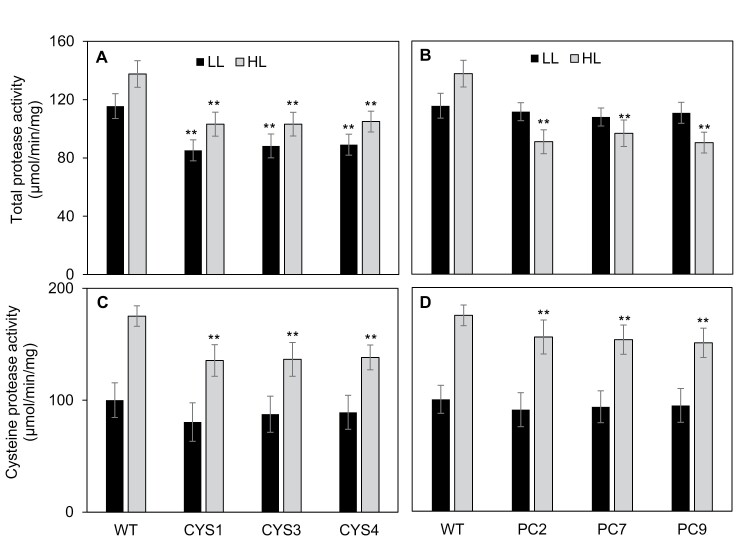
A comparison of the maximal extractable total protease activities and cysteine protease activities from the leaves of the CYS (A and C) and PC (B and D) lines compared with wild-type (WT) plants grown under moderate light (LL) and high light (HL) conditions. The CYS, PC, and WT plants were grown under moderate light (250 μmol m^–2^ s^–1^) for 5 weeks. Thereafter, half of the plants were transferred to HL (800 μmol m^–2^ s^–1^) for 8 h, while the remaining half were maintained for 8 h at 250 μmol m^–2^ s^–1^). The total protease activities and the cysteine protease activities of the leaves were measured between 7 h and 8 h into the photoperiod in all lines. Bars represent means ±SD (*n*=24 individual plants per line). Statistical significance is indicated by asterisks: *P-value ≤0.05.

The total protease activities of the leaves were significantly lower in the CYS lines than in the WT under LL conditions (Fig. 5A, B). Exposure to HL for 8 h significantly increased WT leaf total protease activities (Fig. 5A, B). However, the HL-dependent increases in the leaf protease activities were much lower in the CYS lines than in the WT (Fig. 5A). In addition, no HL-dependent increases in leaf protease activities were observed in the PC lines, which had even lower total protease activities under HL than under LL (Fig. 5B). HL significantly increased the leaf cysteine protease activities of the WT (Fig. 5C, D). However, the HL-dependent increases in leaf cysteine protease activities were, however, significantly lower in the CYS and PC lines than in the WT. While the CYS and PC lines had similar cysteine protease activities under LL (Fig. 5D), the PC lines had higher cysteine protease activities than the CYS lines under HL conditions (Fig. 5C).

**Fig. 6.**
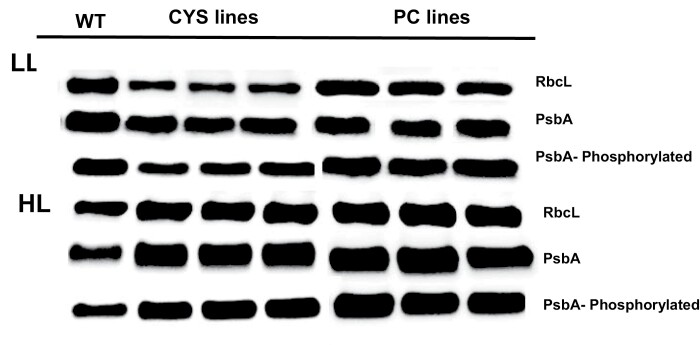
Western blot analysis of the Rubisco large subunit protein (RbcL), the D1 protein. and the phosphorylated form of theD1 protein in the leaves of the CYS and PC lines compared with the wild type (WT). The CYS, PC, and WT plants were grown under moderate light (250 μmol m^–2^ s^–1^) for 5 weeks. Thereafter, half of the plants were transferred to HL (800 μmol m^–2^ s^–1^) for 8 h, while the remaining half were maintained for 8 h at 250 μmol m^–2^ s^–1^. Soluble proteins were extracted from leaves that were harvested 8 h into the photoperiod. Proteins were loaded onto polyacrylamide gels (10 µg of protein per well). After electrophoresis, proteins were transferred to nitrocellulose membranes and analysed with specific antibodies for each protein form.

The abundance of the Rubisco large subunit protein, the D1 protein, and the phosphorylated form of the D1 protein was lower in the leaves of the CYS lines than in those of the WT under LL conditions (Fig. 6). In contrast, the amounts of these photosynthetic proteins were similar in the PC and WT lines (Fig. 6). The amounts of the Rubisco large subunit protein, the D1 protein, and the phosphorylated form of D1 were lower under HL than under LL in the WT plants (Fig. 6). In contrast, the amounts of the photosynthetic proteins were further higher in the Cys and PC lines under HL than under LL (Fig. 6). The HL-dependent increases in the amounts of the chloroplast proteins were most marked in the PC lines.

### Photosynthetic gene expression

**Fig. 7.**
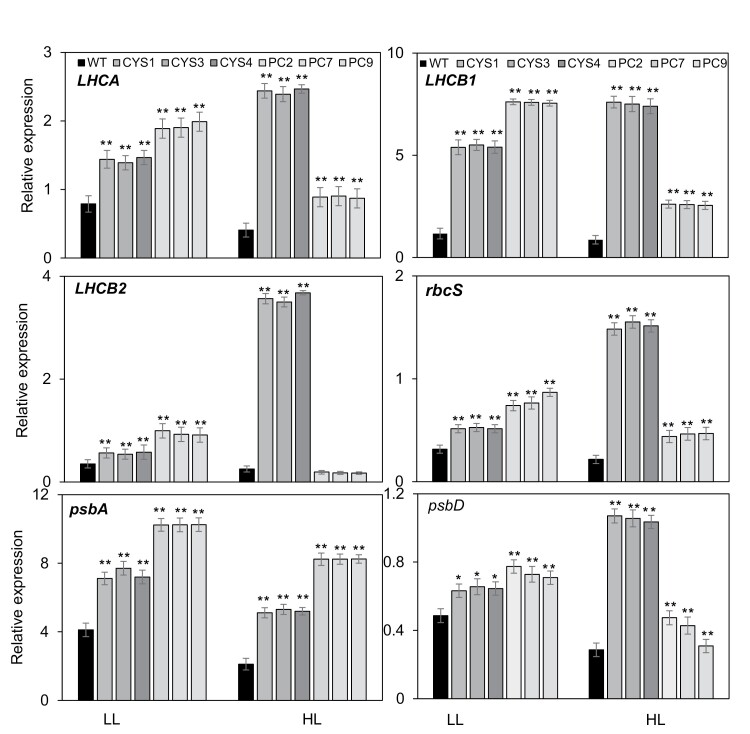
A comparison of the relative abundance of nuclear-encoded chloroplast-targeted photosynthesis transcripts and chloroplast-encoded photosynthesis transcripts in the leaves of CYS, PC, and WT plants grown under moderate light (LL) and high light (HL) conditions. The CYS, PC, and WT plants were grown under moderate light (250 μmol m^–2^ s^–1^) for 5 weeks. Thereafter, half of the plants were transferred to HL (800 μmol m^–2^ s^–1^) for 8 h, while the remaining half were maintained for 8 h at 250 μmol m^–2^ s^–1^. The amounts of transcripts were measured in the leaves of the CYS, PC, and WT plants harvested between 7 h and 8 h into the photoperiod. *LHCA*, light-harvesting Chl *a*-binding protein A; *LHCB1*, light-harvesting Chl *a*/*b*-binding protein B1; *LHCB2*, light-harvesting Chl *a*/*b*-binding protein B2; *rbcS*, the small subunit of Rubisco; *psbA*, PSII D1 protein; *psbD*, PSII D2 protein. Bars represent means ±SD (*n*=24 individual plants per line). Statistical significance is indicated by asterisks: *P-value ≤0.05, **P-value ≤0.01.

We next compared the effects of OC-I expression on the abundance of transcripts encoding chloroplast proteins in the leaves of soil-grown plants. The amounts of transcripts arising from the nuclear-localized genes encoding the light-harvesting Chl *a*/*b*-binding proteins (LHCA, LHCB1, and LHCB2) and chloroplast-localized genes (*rbcS*, *psbA*, and *psbD*) encoding the small subunit of Rubisco, the PSII D1 protein, and the PSII D2 protein, respectively, were increased in the CYS and PC lines compared with the WT under LL conditions (Fig. 7). In general, the amounts of these transcripts were generally higher in the PC lines than in the CYS lines under LL (Fig. 7A). While the amounts of all of these transcripts was even further increased in the CYS lines relative to the WT under HL conditions, the effect of HL was less pronounced in the PC lines than in the CYS lines (Fig. 7). Moreover, in contrast to all other mRNAs measured in this study, the amounts of transcripts encoding the LHCB2 protein were lower in the PC lines than in the WT under HL (Fig. 7). Hence, there are clear differences in the effects of targeting OCI to chloroplasts or the cytosol in the responses of the transcript profile to HL (Fig. 7).

**Fig. 8.**
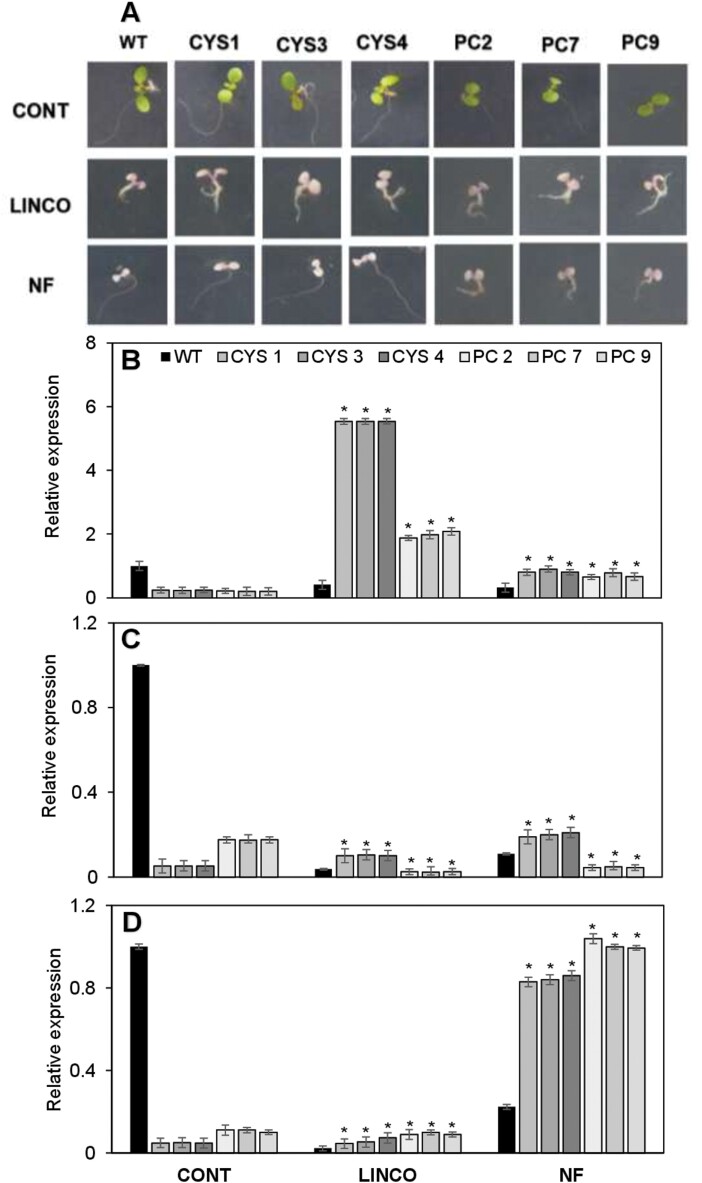
The effects of OC-I expression on chloroplast to nucleus signalling. Wild-type *A. thaliana* (WT) and six independent transgenic lines expressing OC-I either in the cytosol (CYS1, CYS3, and CYS4) or in the chloroplast (PC2, PC7, and PC9) were grown on agar plates under low light (100 μmol m^–2^ s^–1^) with or without lincomycin (LINCO) or norflurazon (NF). (A) Comparisons of representative CYS1, CYS3, CYS4, PC2, PC7, PC9, and WT seedlings grown on agar plates containing either no added inhibitors, LINCO (500 µM), or NF (5 µM) for 7 d. The effect of LINC and NF on the amouns of (B) *LHCA*, (C) *LHCB1*, and (D) *LHCB2* transcripts in the WT, CYS, and PC lines. Bars represent means ±SD (*n*=24 plants per line). Statistical significance as determined by *t*-testing is indicated by asterisks: *P-value ≤0.05.

The data shown in Fig. 7 indicate that the presence of OC-I alters the expression of genes encoding photosynthetic proteins in both the nucleus and chloroplasts. Moreover, differential effects of the OC-I-dependent inhibition of cysteine proteases in chloroplasts on the transcript profile were observed compared with OC-I-dependent inhibition of cysteine proteases in the cytosol. These results suggest that cysteine proteases in both the chloroplasts and the cytosol play roles (and possibly different roles) in chloroplast to nucleus signalling. To explore this possibility further, seedlings were grown for 7 d on agar plates at an irradiance of 100 μmol m^–2^ s^–1^, with or without the chloroplast inhibitors LINCO or NF. The inhibitor treatments led to bleaching of the seedlings (Fig. 8A). Under these LL conditions in the absence of inhibitors, the amounts of *LHCA* (Fig. 8B), *LHCB1* (Fig. 8C), and *LHCB2* (Fig. 8D) transcripts were significantly lower in the CYS and PC lines than in the WT in the absence of inhibitors, but the abundance of *LHCB1* (Fig. 8C) and *LHCB2* (Fig. 8D) mRNAs tended to be higher in the PC lines than in the CYS lines under these conditions.

The LINCO treatment decreased the amounts of the *LHCA* (Fig. 8B), *LHCB1* (Fig. 8C), and *LHCB2* (Fig. 8D) transcripts in the WT but greatly increased the amounts of these mRNAs in the CYS and PC lines, the stimulatory effect being greater in the former than in the latter. In comparison with the effects of LINCO on *LHCA* transcripts in the CYS and PC lines (Fig. 8B), the presence of this inhibitor led to smaller increases in the amounts of *LHCB1* (Fig. 8C) and *LHCB2* (Fig. 8D) transcripts.

The NF treatment decreased the amounts of the *LHCA* (Fig. 8B) *LHCB1* (Fig. 8C), and *LHCB2* (Fig. 8D) transcripts in the WT but greatly increased the amounts of these mRNAs in the CYS lines. In contrast, this inhibitor increased the amounts of *LHCA* (Fig. 8B) and *LHCB2* (Fig. 8D) transcripts in the PC lines, but decreased the as of *LHCB1* (Fig. 8C) transcripts to values lower than those observed in the WT under these conditions.

## Discussion

Chloroplasts belong to the plastid family of plant organelles. Unlike etioplasts that are found in leaves that emerge in the dark and gerontoplasts, which are dominant in senescent leaves, chloroplasts are photosynthetically competent. The different plastid types present in leaves interconvert in response to environmental and developmental triggers as well as in response to biotic and abiotic stresses. These transitions require a complete remodelling of the plastid proteome that transforms the functions and developmental fate of the organelles. Degradation of chloroplast proteins is initiated by chloroplast proteases within the organelle, but the final steps of protein degradation occur outside the plastids through the action of other proteases. Serine, cysteine, aspartic, and metalloprotease expression and activities are detected in senescing leaves, but studies have not revealed the identities of the proteases responsible for chloroplast protein breakdown (Buet *et al.*, 2019). Cysteine proteases, which are important in leaves undergoing rapid protein turnover, are largely located in the central vacuole and other lytic compartments (Buet *et al.*, 2019). In addition, the chloroplast protein complement is reconfigured by regulation of the cytosolic ubiquitin–proteasome system by the CHLORAD-dependent regulation of the TOC machinery at different stages of plant development (Ling *et al.*, 2012, 2019). The data presented here show that cysteine proteases play an important role in determining the chloroplast transcript and proteomic profiles, particularly under different HL conditions.

Cystatins are thiol proteinase inhibitors that are ubiquitously present in plants, animals, and other organisms. The reaction of plant cystatins with cysteine proteases from the C1A papain-like family is highly specific. Cystatins bind to the active site of cysteine proteases as pseudo-substrates, thus preventing cleavage of peptide bonds (Barrett, 1987). While approaches, such as sites, have directed mutagenesis at positively selected amino acid sites have been used to generate cystatin variants with improved inhibitory potency and specificity against specific targets, such as the gut cysteine proteases of herbivorous insects (Goulet *et al.*, 2008), there is no record in the literature of cystatin-dependent inhibition of other types of proteases.

We have not identified which cysteine proteases are involved in the control of chloroplast gene expression and photosynthetic functions, but the results presented here clearly highlight their functional significance, particularly in relation to the chloroplast signalling and responses to HL stress. The study of transgenic plants, in which a cysteine protease inhibitor is expressed in the chloroplasts, allows precise characterization of the functions of putative chloroplast-localized cysteine proteases. In comparison, the consequences of OC-I expression in the cytosol allow characterization of the roles of chloroplast-associated cysteine proteases. We have already characterized the shoot phenotypes of a range of transgenic plants with untargeted expression of OC-I, including tobacco (Prins *et al.*, 2008) and soybean (Quain *et al.*, 2014). The results presented here are the first to characterize transgenic plants in which OC-I expression is targeted to the chloroplasts.

During vegetative growth, the CYS and PC lines had less biomass than the WT line, but the transgenic lines accumulated significantly more biomass during reproductive growth (Fig. 2). These data show that cysteine proteases accelerate shoot biomass production during vegetative growth but they have a negative impact on biomass production after the initiation of flowering. We conclude that the function of cysteine proteases is to accelerate the turnover of chloroplast proteins in leaves after flowering and that OC-I-dependent inhibition of these enzymes has a positive effect on shoot biomass. The slower vegetative growth of the CYS lines agrees with previously published results for OC-I-expressing transgenic tobacco plants (van der Vyver *et al.*, 2003; Prins *et al.*, 2008). The time to flowering was delayed in the CYS and PC lines compared with the WT (Fig. 1A, B). This delay was greatest in the PC lines, suggesting that chloroplast-localized proteins are important in the regulation of flowering and that chloroplast proteases that are blocked by OC-I are important determinants of the timing of flowering (Bryant *et al.*, 2011; Feng *et al.*, 2016).

The targeting of the OC-I transgene to the chloroplasts has a marked effect on leaf pigment contents, an effect that was absent from the CYS lines (Fig. 3A, B). The CYS lines had less chlorophyll (Fig. 3A) and carotenoid pigments (Fig. 3B) than the WT, particularly in the later stages of development. In contrast, the PC rosettes accumulated more leaf pigments than the WT at the later stages of leaf development, suggesting that senescence was delayed in the PC lines. These findings suggest that OC-I-inhibited protease targets in the chloroplasts are involved in pigment biosynthesis and/or degradation. Possible candidates are the degradation of the pigment–protein complexes (Buet *et al.*, 2019; Frank *et al.*, 2019) and the tetrapyrrole biosynthesis pathway (Eckhardt *et al.*, 2004; Tanaka and Tanaka, 2007). Both of these pathways can generate signals, such as singlet oxygen that are involved in chloroplast to nucleus signalling (Rintamäki *et al.*, 2009; Leister, 2019).

Photosynthetic carbon assimilation was better protected against HL-induced inhibition in the CYS and PC lines than in the WT (Fig. 4). This finding may be related to the higher turnover of chloroplast proteins in the WT that is associated with a general increase in leaf protease activities, particularly the cysteine proteases. The amounts of chloroplast proteins (Fig. 6) and the abundance of transcripts (Fig. 7) encoding these proteins were differentially changed in the PC and CYS lines relative to the WT. For example, the Rubisco large subunit and the D1 protein were more abundant in the transgenic lines than in the WT following exposure to HL stress (Fig. 6). Rubisco degradation is a complex process that involves a network of different pathways. Oxidative modification and partial cleavage of the Rubisco protein occurs inside the chloroplasts and thereafter the protein is transported to the vacuole (Ono *et al.*, 2013). The transport of Rubisco and other stromal proteins to vacuolar compartments involves several pathways. Stromal proteins can be degraded by a type of autophagosome known as the Rubisco-containing body (RCB) that allows efficient amino acid recycling (Ishida *et al.*, 2014). Autophagy was found to remove HL-damaged and collapsed chloroplasts in *A. thaliana* (Izumi *et al.*, 2017). Other pathways are independent of autophagy and involve small proteolytic vacuolar compartments (senescence-associated vacuoles) that accumulate during senescence (Martínez *et al.*, 2008). A further pathway is dependent on the chloroplast vesiculation (CV) protein, a process that again involves the formation of vesicles that contain stromal proteins, envelope membrane proteins, and thylakoid membrane proteins (Wang and Blumwald, 2014). In addition, singlet oxygen induces a pathway of chloroplast degradation that is induced by ubiquitination, a process that requires a cytoplasmic ubiquitin E3 ligase called PLANT U-BOX 4 (Woodson *et al.*, 2015). The data presented here implicate chloroplast and cytosolic cysteine proteases in the Rubisco degradation process. These data agree with a recent report concerning the barley HvPAP14 protease, which is involved in the degradation of the large subunit of Rubisco and thylakoid membrane proteins (Frank *et al.*, 2019).

We undertook a phylogenetic analysis to identify Arabidopsis cysteine proteases with homology to HvPAP14. Of the 280 sequences found in the Arabidopsis genome, CEP1 was identified as the closest orthologue to the barley HvPAP14 protein, which was detected in the thylakoid lumen, endoplasmic reticulum, and cytosol (Frank *et al.*, 2019). Like HvPAP14, AtCEP1 carries a C-terminal KDEL motif, which leads to localization in the endoplasmic reticulum, but it has been suggested that AtCEP1 is also active in the cytosol (Howing *et al.*, 2017). Given the observed effects of OC-I expression on the turnover of the D1 protein and the light-harvesting Chl *a*/*b*-binding proteins, the chloroplast-localized cysteine protease should localize to the thylakoid lumen of the chloroplasts. However, further work is required to investigate the intracellular localization and possible roles of AtCEP1 in chloroplast protein turnover.

The amounts of transcripts encoding LHCA, LHCB1, LHCB2, and the D1 protein were increased in the CYS and PC lines relative to the WT (Fig. 7), suggesting that cysteine proteases in the chloroplasts and cytosol may be important for chloroplast to nucleus signalling. We therefore investigated effects on chloroplast to nucleus signalling through the GUN pathway using the chloroplast inhibitors NF and LINCO (Fig. 8) While the amounts of LHCA, LHCB1, and LHCB2 transcripts were higher in the CYS and PC lines than in WT when plants were grown in soil, the opposite was found when the plants were grown on plates in the absence of these inhibitors. The discrepancy in the effects of OC-I on photosynthetic gene expression reported in Figs 7 and 8 is likely to lead to differences in the growth conditions. For example, the plants on plates are grown at a much lower irradiance (100 μmol m^–2^ s^–1^) than those grown on soil (250 μmol m^–2^ s^–1^). Similarly, plants grown on plates are enclosed, with lids limiting the exchange of gases with the atmosphere. However, the observed differences in the amounts of transcripts encoding photosynthetic proteins in the CYS and PC lines under HL suggest that different cysteine proteases are involved in this regulation in the chloroplasts and cytosol.

The GUN pathway of chloroplast to nucleus signalling is impaired by OC-I expression in the chloroplasts and cytosol. As with the amounts of transcripts encoding photosynthetic proteins in the different genotypes grown on soil discussed above, variations in the responses of transcripts that are markers for chloroplast to nucleus signalling were observed in the CYS and PC lines. This finding again suggests that different cysteine proteases and processes may underpin the observed effects. Several components of the GUN pathway have been characterized. For example, GUN1 interacts with the chloroplast chaperone HSC70-1 to promote import of nuclear-encoded chloroplast proteins such as the tetrapyrrole synthesis proteins (Shimizu, *et al.*, 2019). As well as binding to the tetrapyrrole synthesis proteins, GUN1 binds to haem and other porphyrins that affect the flux through the tetrapyrrole synthesis pathway (Shimizu *et al.*, 2019). Further work is required to understand how cysteine proteases are involved in these mechanisms.

In summary, the data presented here show that OC-I expression targeted to the chloroplasts has effects on the HL-induced changes in leaf total protease activities and cysteine protease activities. The level of inhibition of cysteine protease activities achieved with the chloroplast-localized OCI (PC lines) is similar to, but not the same, as that observed in the cytosol-targeted OCI (CYS lines). This suggests that OCI has different targets in both compartments, particularly under HL conditions. Under LL conditions, OC-I expression in the chloroplasts had no detectable effect on the maximum extractable cysteine protease activities from leaves, suggesting that few OC-I-inhibited cysteine proteases are present in chloroplasts under these conditions. However, exposure to HL significantly increased the total protease activities and cysteine protease activities of the WT leaves. Intriguingly, OC-I expression targeted to the chloroplasts resulted in a decrease in both leaf total protease activities and cysteine protease activities, suggesting that cysteine proteases are localized in the chloroplasts, as well as other cellular compartments under HL. Since no cysteine proteases have been identified that have targeting sequences directing the enzyme proteins to the chloroplasts, it is likely that post-translational modifications cause changes in compartmentation, as described for other enzymes (Foyer *et al.*, 2020). The implications of the study are significant in terms of future research on proteases, the regulation of photosynthesis, and the overall reconfiguration of plastid proteins that occurs in response to environmental and developmental triggers. The data presented here demonstrate that cysteine proteases are recruited not only when chloroplasts are converted into gerontoplasts during leaf senescence but also during acclimation to environmental stresses such as exposure to HL. Manipulation of chloroplast-localized cysteine proteases simply using OC-I or similar inhibitors, perhaps under the control of different promoters, may be a useful strategy for the improvement of the efficiency of photosynthesis, particularly in response to changing environmental conditions. Moreover, these data demonstrate that decreasing the stress-induced increases in the turnover of key proteins such as Rubisco has advantages in terms of protection of photosynthesis. Taken together, these data demonstrate that increasing the life span of key proteins by targeting cysteine proteases has advantages in terms of stress tolerance and ultimate productivity.

## Supplementary data

The following supplementary data are available at *JXB* online.

Fig. S1. Examples of the original gels used for Western blot analysis of the Rubisco, D1, and phosphorylated D1 proteins in the leaves of the CYS and PC lines compared with WT Arabidopsis plants grown under moderate light (LL) and high light (HL) conditions. 

Table. S1. List of forward and reverse primers used in the (qRT) PCR analysis

erab101_suppl_Supplemental_Table_S1_and_Figure_S1

## Data Availability

All of the original datasets are available upon request from the authors.
