# Supplementary material for: Papain-like cysteine proteases are required for the regulation of photosynthetic gene expression and acclimation to high light stress
Source: J Exp Bot. 2021 Mar 4;72(9):3441–54. doi: 10.1093/jxb/erab101 (PMC8256631; doi:10.1093/jxb/erab101)
Supplement: erab101_suppl_Supplemental_Table_S1_and_Figure_S1 [file erab101_suppl_supplemental_table_s1_and_figure_s1.pdf]

Supplemental Table S 1.

Genes	Accession	Primers Sequence 5'-3' (Forward / Reverse)
**Actin 2**	At3g18780	GGCTCCTCTTAACCCAAAGG<br>GAGAGAACAGCTTGGATGGC
**Sand family protein (*SAND)***	AT2G28390	AATTAACAGTCCGCAACAGC<br>GACCCAACAGAGTAGAACA
**Photosystem I light harvesting complex A (LHCA)**	At3g61470	TTGGCCATTGAGTTCTTAGCCA<br>AAGCCGACTGTTGCACACAGA
**Light-harvesting chlorophyll a/b-protein 1(LHCB1)**	At1g29930	GGAACGGAGTCAAGTTTGGA<br>CAAAATGCTCTGAGGAA
**Light-harvesting chlorophyll a/b-protein 2(LHCB2)**	At2g05100	AAGTCGTGAATGTACTTATTGGTG<br>GGTGGTGTGGTTCATTAAAGGT
**Ribulose-1,5-bisphosphate carboxylase/oxygenase small subunit (rbcS)**	At1g67090	CCTCCGATTGGAAAGAAGAA<br>TACACAAATCCGTGCTCCAA
**photosystem II protein D1(psbA)**	Atcg00020	GTGGCTGCTCACGGTTATTT<br>CCAAGCAGCCAAGAAGAAGT
**Photosystem I reaction center subunit II (psbD)**	At4g02770	CCGTCCCAAATCCCTCTCCTTC<br>AGAAGACCACCGGTGCTTCCAG

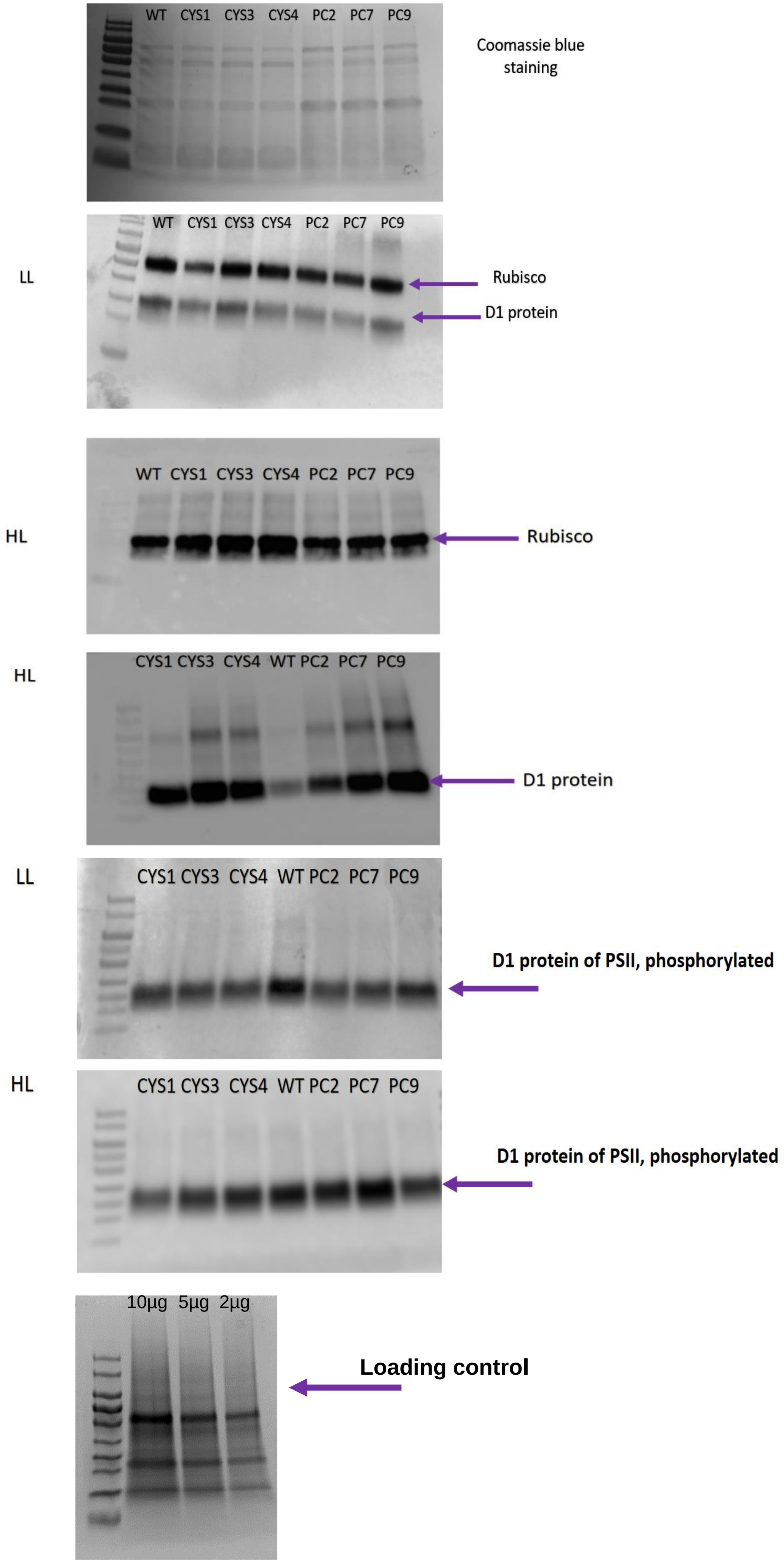
WT CYS1 CYS3 CYS4 PC2 PC7 PC9
Coomassie blue staining
LL
WT CYS1 CYS3 CYS4 PC2 PC7 PC9
Rubisco
D1 protein
HL
WT CYS1 CYS3 CYS4 PC2 PC7 PC9
Rubisco
HL
CYS1 CYS3 CYS4 WT PC2 PC7 PC9
D1 protein
LL
CYS1 CYS3 CYS4 WT PC2 PC7 PC9
D1 protein of PSII, phosphorylated
HL
CYS1 CYS3 CYS4 WT PC2 PC7 PC9
D1 protein of PSII, phosphorylated
10µg 5µg 2µg
Loading control

Supplemental Figure S 1. **Examples of the original gels used for Western-blot analysis of the Rubisco, D1 and phosphorylated D1 proteins in the leaves of the CYS and PC lines compared to WT Arabidopsis plants grown under moderate light (LL) and high light (HL) conditions.** This analysis included a gel stained with Commassie Brilliant Blue and gel of the loading control to ensure appropriate quantitation.
